# Potential of Family Health Strategy Against Cardiovascular Disease

**DOI:** 10.5935/abc.20170187

**Published:** 2017-12

**Authors:** Gilberto Andrade Tavares, José Augusto Soares Barreto-Filho

**Affiliations:** 1 Núcleo de Pós-Graduação em Ciências da Saúde da Universidade Federal de Sergipe (UFS), São Cristóvão, SE - Brazil; 2 Núcleo de Pós-Graduação em Medicina - Universidade Federal de Sergipe, Aracaju, SE - Brazil; 3 Divisão de Cardiologia - Hospital Universitário, Aracaju, SE - Brazil; 4 Clínica e Hospital São Lucas, Aracaju, SE - Brazil

**Keywords:** Family Health / standards, Family Health / education, Cardiovascular Diseases / prevention & control, Myocardial Infarction / mortality, Risk Factors, Epidemiology, Unified Health System / utilization

## Introduction

It is estimated that one out of three adults in the United States of America (USA) has one type of cardiovascular disease (CVD), acute myocardial infarction (AMI) being the major condition. In the USA, more than one million individuals per year are estimated to experience AMI.^1^ In Brazil, in 2011, 384,615 deaths were attributed to CVD.^2^ In 2010, the American Heart Association recommended the assessment of seven metrics related to cardiovascular health (CVH), which could have a great impact on the CVD control. According to the patient adherence to the seven metrics or their control, those metrics (smoking cessation, balanced healthy diet, physical activity practice, and control of body mass, blood pressure, cholesterol and glycemia) were classified as “ideal”, “intermediate” and “poor”, and the goal is to reduce by 20% the deaths from CVD in the USA by 2020.^3^

Several countries have met that recommendation to assess the CVH of their populations. In China, individuals with “ideal” CVH had general mortality 30% lower than that of individuals with “poor” CVH. Regarding mortality from CVD specifically, there was a 39% reduction.^4^ In South Korea, the reduction was of 58% for all-cause mortality, and of 90% for mortality from CVD.^5^ In Brazil, based on data from the 2013 National Health Survey, only 1% of the Brazilian population reached an “ideal” level regarding the seven metrics. When the metrics were considered in isolation, only 3.2% of that population had an “ideal” diet, 23.6% of that population had an “ideal” physical activity practice, and 43.7% of that population had an “ideal” body mass index (BMI). Women had a higher prevalence of “ideal” levels regarding smoking (89.5%). Better levels of blood pressure (77.7%) and total cholesterol (87.3%) were found among men.^6^

Most of the Brazilian population, with or without CVD, has access to healthcare via the Brazilian Unified Health System (SUS). Primary healthcare is the first contact of individuals, families and communities with the SUS, providing healthcare to individuals close to their dwellings, being the first element of the continuous healthcare process.^7^ For primary healthcare to contribute in the prevention and control of chronic diseases, in addition to increasing the amount of individuals cared for, it is necessary to improve access to the system, to encourage the patient’s health self-management, and to improve the training of the healthcare team through education of healthcare providers and support to health managers. It is mandatory to encourage communication with other healthcare levels, facilitating access to specialized diagnosis and treatment services, as well as implantation of efficient systems to improve information recording and use, drug prescription coordination, and follow-up of results.^8^ Therefore, it is necessary to carefully assess the SUS and the complementary healthcare system in Brazil, by use of scientific studies, aiming at the consolidation and construction of an equal, safe, responsive, accessible and efficient healthcare system.^9^

### Family health strategy and cardiovascular health

The Family Health Strategy was chosen to regulate primary healthcare in the SUS. It plays a fundamental role in the first contact of the population with the SUS, and in the healthcare continuation and coordination, and should operate as a base for structuring the healthcare network, counting on the support of diagnosis services, and specialized and hospital care.^10^ However, the CVD care through the Family Health Strategy still has many gaps to be filled in.

Considering the Registry for Hypertensives and Diabetics (HIPERDIA), currently incorporated to the Electronic Basic Healthcare System (e-SUS AB), from a city in the Rio Grande do Sul state, the patients were found to have little blood pressure control and insufficient adherence to treatment.^11^ Among the Family Health Strategy users in the city of Brusque, Santa Catarina state, mean total cholesterol levels were 30% higher than desired. Their low-density lipoprotein-cholesterol levels were 50% above the ideal levels, being, on average, borderline, mainly among women.^12^ In the city of Ribeirão Preto, São Paulo state, among the Family Health Strategy users with diabetes mellitus, glycemia was 60% above the recommended levels, as was glycated hemoglobin.^13^ In the city of Cuiabá, Mato Grosso state, 17.7% of the users (1,402 individuals associated with HIPERDIA) smoked. Most of those patients (81.3%) had an AMI, and 8%, a stroke, and there was no information on the treatment for smoking.^14^

However, there are well-succeeded experiences confirming the potential of the program to fight CVD. Teixeira et al.^15^ assessed the method of nutrition education intervention among female physical activity practitioners in the city of Aracaju, Sergipe state. There was a significant change in dietary habits, number of meals per day and amount of food consumed favoring the intervention group. In addition, there was a mean IMC reduction by 11.19 kg/m^2^ (p < 0.05).^15^ Rocha et al.^16^ developed, in inner Bahia state, a physical activity program in the Family Health Strategy. The intervention group showed a significant reduction in mean systolic blood pressure by 47.3 mm Hg (p = 0.003), in blood glucose levels, by 33.4-mg/dL, and in BMI, by 1.1-kg/m^2^ (p < 0.001).^16^

## Conclusions and potentials

Considering the global CVD epidemic, especially in developing countries, we believe that the Family Health Strategy can play a central role in both promoting better CVH and fighting CVD. The scarce literature on the topic indicates that the control of biological and behavioral factors relating to CVH by the Family Health Strategy is far from ideal. However, some well-succeeded experiences point to the potential of Family Health Strategy to fight CVD.

In the current phase of the program, Family Health Strategy needs to be better investigated from the scientific evidence viewpoint. Healthcare Research would be useful. Encouraging studies to generate evidence on the real impact of Family Health Strategy on Brazilian cardiovascular public health could promote constant systemic improvement in the program, in addition to supporting more effective and efficient health policies to reduce the perspective of CVD increase in Brazil.
